# Factors shaping giraffe behavior in U.S. zoos: A multi-institutional study to inform management

**DOI:** 10.1371/journal.pone.0324248

**Published:** 2025-05-29

**Authors:** Jason D. Wark, Katherine A. Cronin

**Affiliations:** Animal Welfare Science Program, Lincoln Park Zoo, Chicago, Illinois, United States of America; University Centre Sparsholt, UNITED KINGDOM OF GREAT BRITAIN AND NORTHERN IRELAND

## Abstract

Giraffes are one of the most commonly housed megafauna in zoos. Variation across zoos (e.g., geographic location, housing, and husbandry), may be expected to influence giraffe behavior. However, past studies have typically focused on a small number of zoos, limiting generalizable conclusions. Here, we expand upon a recent report on the behavior patterns of zoo-housed giraffes to evaluate the influence of several factors on giraffe behavior. Data were recorded on 66 individuals housed across 18 zoos over a one-year period (n = 8,330 10-min observation sessions). Generalized linear mixed models were used to analyze influences on the following behaviors: browsing, extractive foraging, other feeding, ruminating, oral stereotypies, inactive, and locomotion. Behaviors were first compared across outdoor and indoor housing conditions, then models were built for each behavior and housing condition to evaluate how multiple factors influenced behavior: organizational (habitat size, herd size, presence of public feeding opportunities); environmental (temperature, weather); temporal (time of day); and individual (age, sex). Behavioral differences between outdoor and indoor conditions were observed but were minimal. Overall, environmental and temporal factors influenced giraffe behavior the most, but all factors significantly influenced at least one behavior. Several findings are relevant for giraffe management. For example, giraffes living at zoos with public feeding opportunities spent less time browsing, more time in other feeding behaviors, and showed a trend of increased inactivity, suggesting these programs may negatively influence giraffe behavior. Oral stereotypies were negatively correlated with the time spent browsing or extractive foraging and temperature. This supports husbandry efforts to increase natural foraging behaviors in giraffes and highlights the need to better understand thermal comfort. The amount of outdoor space available was not associated with the time spent locomoting, countering common assumptions. Overall, these findings can begin to inform management decisions and guide additional investigations to identify specific, evidence-based husbandry recommendations.

## Introduction

Many regional zoological associations represent a network of organizations focused on the cooperative population management and care of species. As such, individuals of a given species may live in a variety of settings as they are housed across different organizations. Although differences between organizations, such as their geographic locations, size of habitats, and size of social groups, may be expected to influence animal behavior, and potentially welfare, these factors are difficult to assess given the challenges associated with the coordinated multi-institutional approach needed [1].

Within the Association of Zoos and Aquariums (AZA), multi-institutional studies have been conducted that evaluated broad differences in housing and husbandry across a large number of organizations for different species, including black rhinos (60 individuals across 23 zoos) [2], clouded leopards (72 individuals across 12 zoos) [3], polar bears (55 individuals across 20 zoos) [4], and elephants (89 individuals across 39 zoos) [5]. Given their robust sample sizes, these studies were able to reveal effects of the zoo environment not possible in smaller studies, such as the finding that smaller enclosures for black rhinos were associated with more dominant behavior profiles [2], a negative relationship between enclosure height and glucocorticoids for clouded leopards [3], larger social groups of polar bears being associated with decreased pacing [4], and a greater number of social groups of elephants being related to increased walking [6]. Recently, we shared a descriptive account of the behavior patterns of giraffes from a large, multi-institutional study (66 individuals across 18 zoos) [7]. Here, we expand on this exploratory study to evaluate how aspects of the zoo environment affect giraffe behavior.

Giraffes are one of the most commonly housed megafauna in zoos, with over 500 individuals living across more than 100 accredited organizations in the AZA and many more globally. They have also been a frequent focus of multi-institutional studies [8–14]. Several early studies found few behavioral differences in giraffes between zoos [8,10]. This led Bashaw [10] to suggest that giraffe behavior was broadly consistent across zoos. In fact, in a large study of overnight behavior that included 63 giraffes across 13 zoos, Burger et al. [13] found no significant effects based on habitat size or herd size, questioning the impact that organization plays in giraffe behavior. However, research by Orban et al. [11] did identify some behavioral differences in giraffes between zoos with and without public giraffe feeding opportunities, demonstrating that husbandry or programmatic decisions can influence giraffe behavioral expression. Therefore, the degree to which giraffe behavior is influenced by aspects of the zoo environment remains an open question.

Research in zoos has identified individual and seasonal effects on behavior [12–14]. For example, feeding and foraging behaviors have been found to vary based on season, with increased foraging in indoor winter habitats compared to the summer [12,14]. Individual behavioral differences have also been documented in giraffes living in the wild. For example, studies have described differences in foraging behavior between males and females, with females spending more time feeding and less time ruminating than males [15–18]. This difference has been attributed to the larger gut of males, enabling them to feed at a faster rate and spend longer processing their food [19]. Age-related differences in behavior have also been noted, with subadults spending less time feeding and ruminating than adults, which was suggested to reflect the subadults’ need for vigilance to avoid aggression [17]. In zoos, one study identified a trend of more time spent feeding by females as compared to males [9], but another study failed to identify sex differences in feeding [12]. Currently, it is unclear what role sex and age play in the behavior of zoo-housed giraffes.

Time of day is an additional factor likely to influence giraffe behavior. Giraffes in the wild have documented peaks in feeding during the morning and evening with increased time resting and ruminating during midday to early evening [16,18,20]. This pattern has been attributed to minimizing heat load by avoiding active behaviors during the hottest parts of the day [16]. A similar temporal pattern has also been described in zoos [21], which may relate to temperature or husbandry schedules.

Here, we draw on the power of a multi-institutional study with a robust sample size to address open questions on the influence of housing and environment on giraffe behavior in zoos. Recently, the behavior patterns of giraffes from 18 zoos were described [7]. Here, we build off that work to identify significant factors influencing giraffe behavior. Our primary focus is on organizational factors, including habitat size, herd size, and whether a zoo offers public feeding experiences. In addition, we consider environmental (i.e., temperature, weather), temporal (time of day), and individual (e.g., age, sex) factors that could influence behavior. Although not exhaustive, these factors include core components of care and population structure that vary across zoos. Understanding the role these factors can play on giraffe behavior is a critical first step toward developing evidence-based husbandry recommendations that can advance best practices in the care of giraffes.

## Methods

### Ethical statement

This study was approved by Lincoln Park Zoo’s Research Committee and the institutional research review boards at each participating zoo.

### Subjects and housing

Subjects for this study included 66 giraffes (26 males, 40 females) housed across 18 zoos accredited by the Association of Zoos and Aquariums. These zoos represented organizations of varying size and geographic location in the United States. Summary information for each zoo is provided in Table 1. Giraffes at each zoo were observed in their outdoor habitat. In addition, giraffes were observed in indoor habitats where possible. See Wark and Cronin [7] for more details on the selection of study subjects and housing.

**Table 1:** Summary of animals and housing per zoo.

Zoo ID	Study Subjects				Total Herd Size	Outdoor Habitat Size (m^2^)	Indoor Habitat Size (m^2^)	Public Feeding
	Adult Males	Adult Females	Subadult^1^ Males	Subadult^1^ Females				
Zoo 1	0	2	2	0	4	3159	139.4	Yes
Zoo 2	1	1	1	1	4	1393	325.2	Yes
Zoo 3	2	1	0	1	4	2973	697	Yes
Zoo 4	0	3	0	0	5	4831	0	No
Zoo 5	0	2	0	1	5	6612	763	Yes
Zoo 6	2	0	0	0	2	464	0	Yes
Zoo 7	1	2	0	0	3	1115	85	No
Zoo 8	1	3	0	1	10	5832	0	Yes
Zoo 9	1	3	0	0	4	55741	0	No
Zoo 10	2	0	0	1	4	9848	93	Yes
Zoo 11	2	0	0	0	2	1858	268	Yes
Zoo 12	0	2	0	1	9	836	93	Yes
Zoo 13	0	2	1	0	4	2323	269	Yes
Zoo 14	1	2	1	2	6	3344	0	Yes
Zoo 15	1	1	1	0	8	4552	697	Yes
Zoo 16	0	2	0	1	6	263043	0	No
Zoo 17	4	2	1	1	17	40468	557	Yes
Zoo 18	1	2	0	0	3	7156	167	Yes

^1^Individuals under 4 years old were considered subadult based on the age of sexual maturity in giraffes [22].

### Data collection

Behavior data collection and project coordination were conducted with ZooMonitor [23] using the ZooMonitor Community collaborative platform. Data were collected for approximately one year at each participating zoo, starting in January 2022 and continuing to March 2023. We aimed for a minimum of 45 weeks of observation per individual. However, due to the inclusion of several giraffes during the study and the death of one focal individual, fewer weeks of observation (i.e., < 45) were available for some giraffes (n = 6). These individuals were retained in the analyses as decreased observation time was not expected to influence the overall results.

Giraffe behavior was recorded by trained observers at each zoo using the ZooMonitor app [24]. Ten-minute observation sessions were conducted during daytime hours (6:00–18:00) and approximately balanced between morning and afternoon. Based on observer schedules and animal access, most observation sessions occurred between 9:00 and 15:00 (96%). Behavior was recorded at one-min. intervals using instantaneous point sampling methods. A full ethogram was followed that included inactive behaviors (standing and sitting), feeding and foraging behaviors (browsing, extractive foraging, other feeding, ruminating), locomotion, stereotypic behaviors (tongue play, repetitive licking, pacing), and social behaviors (affiliative, sexual, agonistic) [25]. See S2 Table for the definitions of behaviors considered in this study. Behaviors were recorded as mutually exclusive states. Observations were conducted either live (via in-person or real-time video stream) or using pre-recorded video footage. Observers were primarily volunteers and interns. In addition, observations were also recorded by researchers and animal keepers. To ensure consistent data collection across observers, all observers completed a three-step training and testing process prior to data collection that included: 1) an online quiz with brief video snippets for identification; 2) a virtual reliability test on a 10-min video; and 3) an in-person reliability test with a project lead at each zoo [26]. Reliability tests required a mean percent agreement of 85% or better to pass. See Wark and Cronin [7] for more details.

### Data analysis

Given the large differences in characteristics between indoor and outdoor habitats, behavior data collected indoors and outdoors were analyzed separately. Sessions in which giraffes had simultaneous access to indoor and outdoor areas were relatively rare (n = 866; 9% of the total dataset). As simultaneous access between indoor and outdoor spaces was rare and was often given in response to inclement weather or for medical reasons, these data were excluded from analysis. Data from indoor-only sessions at two zoos (Zoo 8 and Zoo 11) were also excluded as this housing condition was only noted once for Zoo 8 and twice for Zoo 11. To account for changes in animal visibility across zoos and throughout the year, data were analyzed as a proportion of time visible. Sessions with less than five visible intervals (i.e., less than half the observation session) were excluded from the analysis to prevent artificially inflated proportion values [27].

Giraffe behavior was analyzed using generalized linear mixed models from the glmmTMB package [28] in R [29]. Models were constructed for the following focal behaviors: browsing, extractive foraging, other feeding, ruminating, oral stereotypy (repetitive licking + tongue play), inactive (standing + sitting), and locomotion. These were the most commonly observed behaviors in this study and were selected based on their presumed importance for giraffe welfare (see [7]). Models were generated using the beta binomial distribution with logit link function to account for overdispersion in the data (i.e., greater variance in the data than predicted by the model; [30]). The beta binomial family models the data as a binomial process of “success” and “failures” using the beta distribution with an additional overdispersion parameter (θ) calculated to model excess variation in the data. This distribution has been shown appropriate for biological data when the number of random effect levels is greater than five [31]. Here, the count of intervals of each focal behavior per session was recorded as “successes” and the count of intervals for other visible behaviors (i.e., excluding animal not visible and behavior obscured) as “failures”. An initial model was first constructed including both outdoor and indoor observations sessions to evaluate the effect of housing condition. Subsequent behavior models were then constructed separately for sessions with outdoor-only and indoor-only access. Fixed effects considered in this study are described in Table 2. All behavior models included the following fixed factors: age, sex, time, and habitat size. For outdoor-only models, temperature, weather and public feeding were included, given their relative potential importance for this housing condition. Similarly, for models of oral stereotypy behavior, the count of intervals feeding/ foraging and ruminating were included as a fixed effect based on past research suggesting a relationship between these behaviors (see Discussion). Temperature and habitat size were scaled to allow comparison of model coefficients across covariates (see Table 2). In addition, outdoor habitat size was log transformed prior to scaling to account for the large variation in outdoor habitat sizes across zoos and assumed non-linear relationship with behavior. A random intercept of individuals nested within zoos was included in models to account for issues related to repeated measures (i.e., pseudoreplication) and the hierarchical structure of the data (e.g., zoo-specific differences in husbandry).

**Table 2:** Overview of fixed factors considered in behavior models.

Type	Fixed Factor	Formula/Options	Model
Organizational	Outdoor Habitat Size	(Log(Habitat Size (m^2^)) – Mean (Log(Habitat Size (m^2^))))/ Std Dev(Log(Habitat Size (m^2^)))	Outdoor only
	Indoor Habitat Size	(Habitat Size (m^2^)) – Mean (Habitat Size (m^2^))))/Std Dev(Habitat Size (m^2^)))	Indoor only
	Herd Size	Sum of number of animals	All
	Public Feeding	Yes/No	Outdoor only
Environmental	Temperature	(Temperature (°C) – Mean(Temperature (°C)))/Std Dev(Temperature (°C))	Outdoor only
	Weather	Sunny/ Rainy/ Cloudy	Outdoor only
Temporal	Time	Morning (< 11:30 AM)/Midday (11:30 AM to 1:30 PM)/ Afternoon (> 1:30 PM)	All
Individual	Age	Subadult (0–4 y.o.)/Adult (>4 y.o.)	All
	Sex	Male/Female	All
	Feeding/ Foraging Count	Sum of intervals with feeding/foraging behaviors	Oral stereotypy only
	Ruminating Count	Sum of intervals with feeding/foraging behaviors	Oral stereotypy only

Model fits were evaluated by examining the quantile-quantile plot of residuals and testing residuals for normality and outliers using the DHARMa package in R [32]. Collinearity was assessed by calculating variance inflation factors (VIF) using the check_collinearity function in the performance package in R [33]. The VIF score was less than two for all fixed effects, indicating multicollinearity was not present in the models [34]. Significant effects were evaluated using the ANOVA function in the car package of R [35] to calculate Wald type 2 Analysis of Deviance metrics. When significant main effects were identified, estimated marginal means were calculated using the emmeans function in the emmeans package in R [36], with a Tukey adjustment for post-hoc multiple comparisons.

Given previous research has suggested a relationship between oral stereotypies and feeding-related behaviors, therefore a correlation test between these behaviors was conducted. As a Shapiro-Wilk normality test indicated most of these behaviors were non-normal, a Spearman’s rank correlation test was performed on individual mean values.

Significance was considered when α ≤ 0.05 and a trend to significance was noted when α ≤ 0.10.

## Results

**Fig 1:**
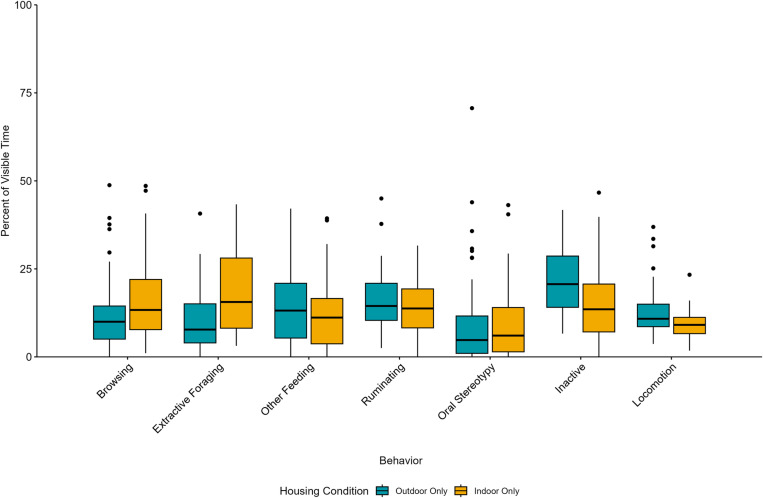
The behavior patterns of giraffes observed in this study by housing access. The boxplot displays the percent of visible time for each individual’s mean behavior value as boxes representing the 25th and 75th percentiles, the median indicated as a horizontal line, whiskers representing the largest value within 1.5 times the interquartile range, and dots to indicate individual outliers defined as values above and below 1.5 times the interquartile range.

A total of 8,330 observation sessions were analyzed in this study (Outdoor: n = 6507; Indoor: n = 1823; S1 Dataset). Observations were recorded primarily live in-person (93%). See S3 Table for a detailed summary of the number of sessions recorded per zoo by housing condition and time of day. Behavior patterns differed by indoor and outdoor housing condition (Fig 1). Housing condition was a significant predictor in generalized linear mixed models for all study behaviors except ruminating (browsing: χ^2^ = 76.583, df = 1, *p* < 0.001; extractive foraging: χ^2^ = 99.355, df = 1, *p* < 0.001; other feeding: χ^2^ = 56.606, df = 1, *p* < 0.001; ruminating: χ^2^ = 0.577, df = 1, *p* = 0.447; oral stereotypy: χ^2^ = 8.875, df = 1, *p* = 0.003; inactive: χ^2^ = 52.565, df = 1, *p* < 0.001; locomotion: χ^2^ = 42.708, df = 1, *p* < 0.001).

Generalized linear mixed models were evaluated for each behavior while indoors and outdoors. Table 3 provides an overview of significant effects across models. Time of day was a significant predictor of all seven behaviors evaluated and environmental factors were significant for six of the seven behaviors. Organizational and individual factors were each significant for four of the seven behaviors. Full model results are provided below per behavior and summarized in S4 Table.

**Table 3:** Overview of *p*-values^a^ for predictor variables^b^ from behavior models for each housing condition.

Behavior/ Housing	Habitat Size	Herd Size	Public Feeding	Time	Temperature	Weather	Age	Sex	Feeding Count	Ruminating Count
Browsing										
Outdoor	**<0.001** **(-)**	0.331	**<0.001** **(↓ public feeding)**	**<0.001** **(↑ morning)**	0.594	*0.073* *(n.s.)*	*0.069* *(↑ subadults)*	**0.002** **(↑ males)**	n.a.	n.a.
Indoor	0.373	**0.045 (-)**	n.a.	**<0.001** **(↓ morning)**	n.a.	n.a.	**0.044** **(↑ subadults)**	0.596	n.a.	n.a.
Extractive Foraging										
Outdoor	0.661	0.888	0.157	**0.001** **(↑ morning)**	**<0.001** **(-)**	0.618	0.596	**0.007** **(↑ males)**	n.a.	n.a.
Indoor	**0.005** **(-)**	**0.029 (+)**	n.a.	0.207	n.a.	n.a.	0.363	0.658	n.a.	n.a.
Other Feeding										
Outdoor	**0.033** **(+)**	*0.077* *(-)*	**<0.001** **(↑ public feeding)**	**<0.001** **(↓ afternoon)**	**0.002** **(+)**	**0.009** **(↑ rainy)**	0.242	0.725	n.a.	n.a.
Indoor	**0.014** **(+)**	*0.097 (-)*	n.a.	**0.034** **(↑ afternoon)**	n.a.	n.a.	*0.061* *(↑ subadults)*	0.718	n.a.	n.a.
Ruminating										
Outdoor	0.494	0.956	0.372	**0.045** **(↓ morning)**	**0.008** **(-)**	**0.023** **(↑ rainy)**	*0.088* *(↓ subadults)*	**0.025** **(↓ males)**	n.a.	n.a.
Indoor	0.167	0.705	n.a.	0.263	n.a.	n.a.	0.290	*0.097* *(↓ males)*	n.a.	n.a.
Oral Stereotypy										
Outdoor	0.122	0.979	0.726	**0.030** **(↑ midday)**	**<0.001** **(-)**	0.234	0.306	0.213	**<0.001** **(-)**	**<0.001** **(-)**
Indoor	0.632	**0.050 (+)**	n.a.	0.122	n.a.	n.a.	0.594	0.225	**<0.001** **(-)**	**<0.001** **(-)**
Inactive										
Outdoor	*0.089* *(+)*	0.441	*0.067* *(↑ public feeding)*	**<0.001** **(↑ afternoon)**	**<0.001** **(+)**	0.848	0.905	0.621	n.a.	n.a.
Indoor	0.215	**0.040 (-)**	n.a.	*0.073* *(↑ morning)*	n.a.	n.a.	0.985	0.233	n.a.	n.a.
Locomotion										
Outdoor	0.942	**0.048** **(-)**	0.839	**<0.001** **(↑ afternoon)**	**<0.001** **(-)**	**<0.001** **(↑ cloudy)**	0.225	0.254	n.a.	n.a.
Indoor	0.244	**0.005 (-)**	n.a.	**<0.001** **(↑ morning)**	n.a.	n.a.	0.655	**0.040** **(↑ males)**	n.a.	n.a.

^a^Significant effects (*p* ≤ 0.05) are shown in bold, and trends (*p* ≤ 0.10) are shown in italics.

^b^For continuous predictors, negative relationships are indicated with a “-” and positive with a “+” in parentheses below the p-value.

### Browsing

**Fig 2:**
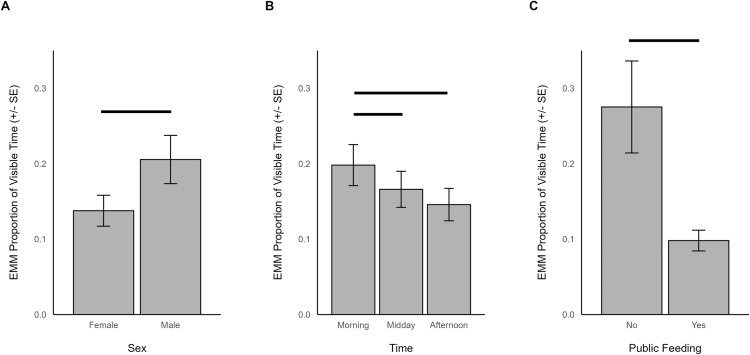
The estimated marginal means for significant predictors of browsing behavior when giraffes were housed outdoors. Significant differences are indicated with a line.

For times when giraffes were housed outdoors, the proportion of intervals of browsing behavior was significantly influenced by sex (χ^2^ = 9.631, df = 1, *p *= 0.002), time (χ^2^ = 32.953, df = 2, *p* < 0.001), outdoor habitat size (χ^2^ = 17.460, df = 1, *p* < 0.001), and whether the zoo offered public feeding programs (χ^2^ = 12.754, df = 1, *p* < 0.001). Specifically, browsing was more commonly observed in males (EMM = 0.206, SE = 0.032) than females (EMM = 0.138, SE = 0.020; Fig 2A), occurred more in the morning (EMM = 0.198, SE = 0.027) than midday (EMM = 0.166, SE = 0.024; z = 3.499, p = 0.001) or afternoon (EMM = 0.146, SE = 0.021; z = 5.559, p < 0.001; Fig 2B), was more frequent for giraffes in smaller habitats (β = -0.643, SE = 0.154), and occurred more in zoos that did not offer public feeding (EMM = 0.275, SE = 0.061) compared to zoos with public feedings (EMM = 0.098, SE = 0.014; Fig 2C). In addition, a trend was noted of more time spent browsing by subadults (EMM = 0.191, SE = 0.032) than adults (EMM = 0.149, SE = 0.020; χ^2^ = 3.301, df = 1, *p* = 0.069).

**Fig 3:**
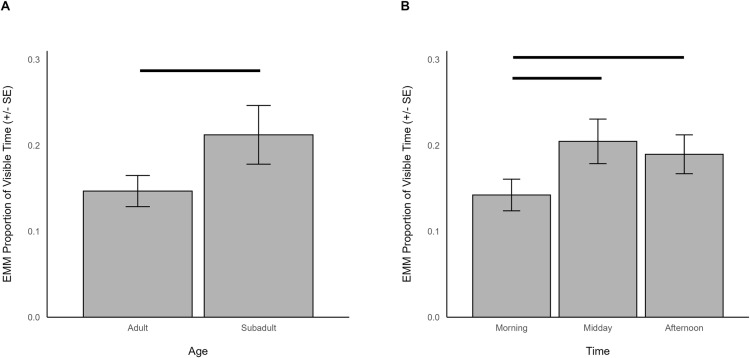
The estimated marginal means for significant predictors of browsing behavior when giraffes were housed indoors. Significant differences are indicated with a line.

When indoors, browsing was significantly predicted by age (χ^2^ = 4.054, df = 1, *p *= 0.044), time (χ^2^ = 17.522, df = 2, *p *< 0.001), and herd size (χ^2^ = 4.012, df = 1, *p *= 0.045). Browsing when indoors was observed more in subadults (EMM = 0.212, SE = 0.034) than adults (EMM = 0.147, SE = 0.018; Fig 3A), occurred less in the morning (EMM = 0.142, SE = 0.018) than midday (EMM = 0.205, SE = 0.026; z = -3.848, p < 0.001) or afternoon (EMM = 0.190, SE = 0.023; z = -3.147, p = 0.005; Fig 3B), and decreased with increasing herd sizes (β = -0.060, SE = 0.030).

### Extractive feeding

**Fig 4:**
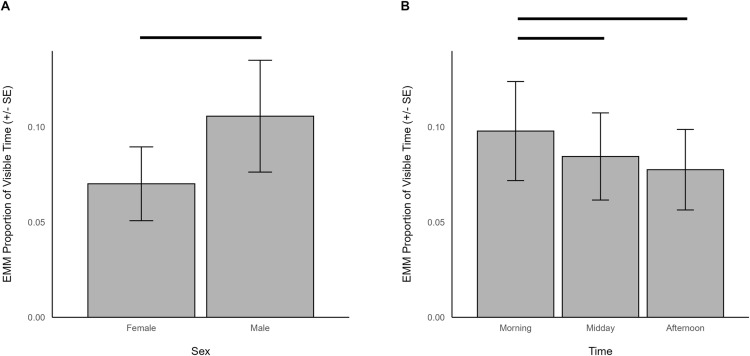
The estimated marginal means for significant predictors of extractive foraging behavior when giraffes were housed outdoors. Significant differences are indicated with a line.

For outdoor-only sessions, the proportion of visible intervals during which giraffes displayed extractive foraging behavior was significantly predicted by sex (χ^2^ = 7.177, df = 1, *p *= 0.007), time (χ^2^ = 13.352, df = 2, *p *= 0.001), and temperature (χ^2^ = 32.933, df = 1, *p *< 0.001). Extractive foraging when outdoors was observed more frequently by males (EMM = 0.106, SE = 0.029) than females (EMM = 0.070, SE = 0.019; Fig 4A), more commonly occurred during the morning (EMM = 0.098, SE = 0.026) than midday (EMM = 0.084, SE = 0.022; z = 2.352, p = 0.049) or afternoon (EMM = 0.078, SE = 0.021; z = 3.478, p = 0.001; Fig 4B), and decreased with increasing temperature (β = -0.258, SE = 0.045).

When giraffes were housed indoors, extractive foraging was predicted by herd size (χ^2^ = 4.747, df = 1, *p *= 0.029) and indoor habitat size (χ^2^ = 7.978, df = 1, *p* = 0.005). The time spent extractive foraging was greater for giraffes living in larger herds (β = 0.094, SE = 0.043) and less for giraffes with smaller indoor habitats (β = -0.564, SE = 0.200).

### Other feeding

**Fig 5:**
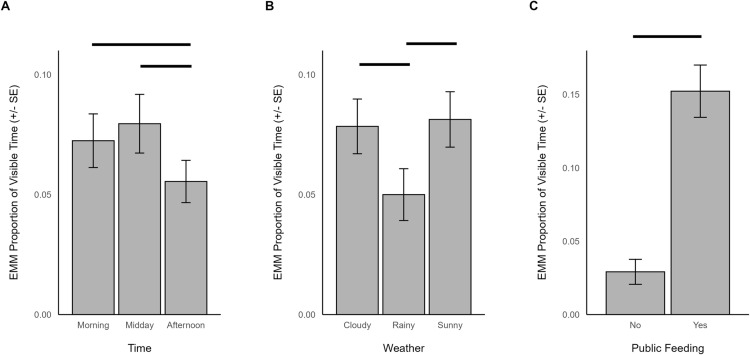
The estimated marginal means for significant predictors of other feeding behavior when giraffes were housed outdoors. Significant differences are indicated with a line. Note the difference in y-axis range between plots.

During times housed outdoors, other feeding behavior was significantly influenced by time (χ^2^ = 36.184, df = 2, *p *< 0.001), temperature (χ^2^ = 9.479, df = 1, *p *= 0.002), outdoor habitat size (χ^2^ = 4.559, df = 1, *p *= 0.033), weather (χ^2^ = 9.504, df = 2, *p *= 0.009), and whether a zoo had public feeding opportunities (χ^2^ = 28.280, df = 1, *p *< 0.001). Specifically, other feeding behaviors occurred less in the afternoon (EMM = 0.055, SE = 0.009) than in the morning (EMM = 0.072, SE = 0.011; z = 4.518, p < 0.001) or midday (EMM = 0.079, SE = 0.012; z = 5.911, p < 0.001; Fig 5A), increased with increasing temperature (β = 0.113, SE = 0.037), was more frequent for giraffe in larger outdoor habitats (β = 0.303, SE = 0.142), was lower when it was rainy (EMM = 0.050, SE = 0.011) compared to when it was sunny (EMM = 0.081, SE = 0.011; z = -3.052, p = 0.006) or cloudy (EMM = 0.078, SE = 0.011; z = 2.790, p = 0.006; Fig 5B), and occurred more in zoos that offered public feeding programs (EMM = 0.152, SE = 0.018) than those that did not (EMM = 0.029, SE = 0.008; Fig 5C).

**Fig 6:**
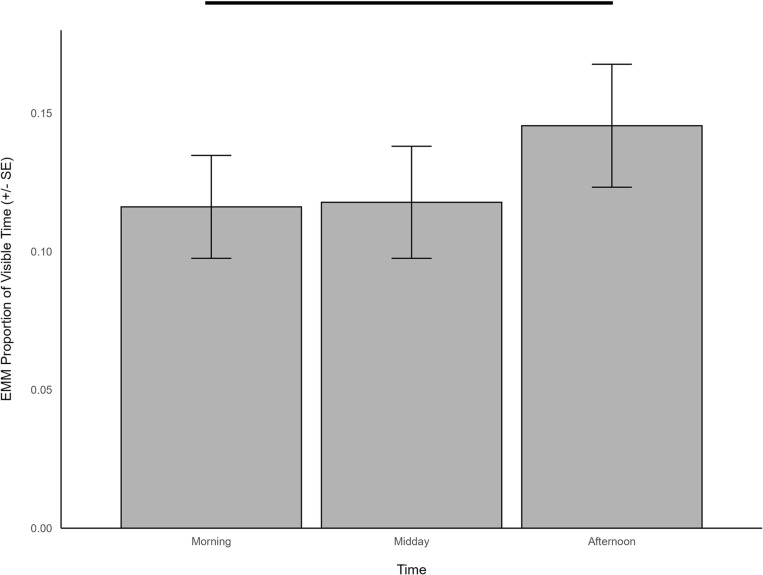
The estimated marginal means for significant predictors of other feeding behavior when giraffes were housed indoors. Significant differences are indicated with a line.

For indoor-only sessions, other feeding behaviors were predicted by time of day (χ^2^ = 6.752, df = 2, *p *= 0.034) and herd size (χ^2^ = 6.009, df = 1, *p *= 0.014). Giraffes spent more time engaged in other feeding behaviors in the afternoon (EMM = 0.145, SE = 0.022) than morning (EMM = 0.116, SE = 0.019; z = -2.434, p = 0.040; Fig 6). Giraffes with larger indoor habitats spent more time in other feeding behaviors (β = 0.466, SE = 0.190). There was also a trend of age (χ^2^ = 3.501, df = 1, *p *= 0.061), with more time spent on other feeding behaviors by subadults (EMM = 0.138, SE = 0.022) compared to adults (EMM = 0.115, SE = 0.018).

### Ruminating

**Fig 7:**
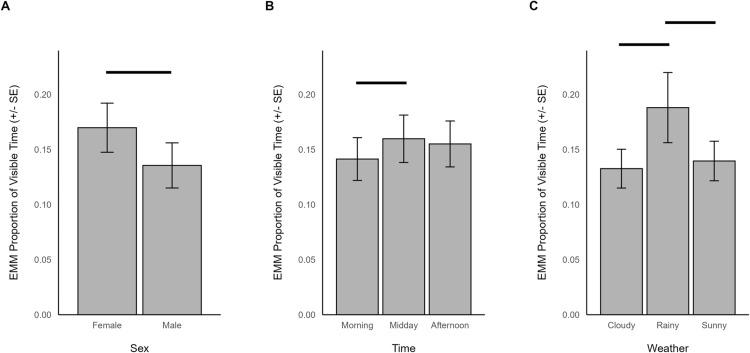
The estimated marginal means for significant predictors of ruminating behavior when giraffes were housed outdoors. Significant differences are indicated with a line.

Ruminating during outdoor-only sessions was significantly predicted by sex (χ^2^ = 5.031, df = 1, *p *= 0.025), time (χ^2^ = 6.189, df = 2, *p *= 0.045), temperature (χ^2^ = 7.117, df = 1, *p *= 0.008), and weather (χ^2^ = 7.500, df = 2, *p *= 0.0235). When outdoors, ruminating was more frequent in females (EMM = 0.170, SE = 0.022) than males (EMM = 0.136, SE = 0.020; Fig 7A), lower in the morning (EMM = 0.141, SE = 0.019) than midday (EMM = 0.160, SE = 0.021; z = -2.356, p = 0.048; Fig 7B), decreased with increasing temperature (β = -0.100, SE = 0.037), and was more common when it was rainy (EMM = 0.188, SE = 0.032) compared to times when it was cloudy (EMM = 0.133, SE = 0.018; z = -2.695, p = 0.019) or sunny (EMM = 0.140, SE = 0.018; z = 2.359, *p* = 0.048; Fig 7C).

When indoors, ruminating was not significantly predicted by any factors. A trend to significance was noted for sex (χ^2^ = 2.749, df = 1, *p *= 0.097), with more ruminating being displayed by females (EMM = 0.133, SE = 0.023) than males (EMM = 0.105, SE = 0.020).

### Oral stereotypies

**Fig 8:**
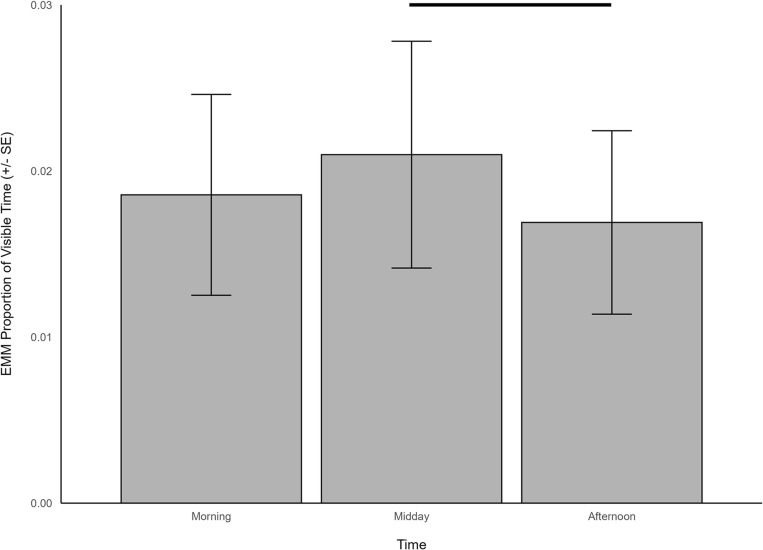
The estimated marginal means for significant predictors of oral stereotypic behavior when giraffes were housed outdoors. Significant differences are indicated with a line.

When giraffes were housed outdoors, the expression of oral stereotypies was predicted by time (χ^2^ = 7.027, df = 2, *p *= 0.030), temperature (χ^2^ = 12.570, df = 1, *p *< 0.001), and the count of intervals spent feeding/ foraging (χ^2^ = 844.086, df = 1, *p *< 0.001) and ruminating (χ^2^ = 379.056, df = 1, *p *< 0.001). Oral stereotypies were observed to be higher midday (EMM = 0.021, SE = 0.007) compared to the afternoon (EMM = 0.017, SE = 0.006; z = 2.587, *p* = 0.026; Fig 8), and had a negative relationship with temperature (β = -0.170, SE = 0.048), feeding/foraging (β = -0.387, SE = 0.013), and ruminating (β = -0.362, SE = 0.018) such that oral stereotypies tended to increase as temperature, feeding/foraging, and ruminating decreased.

The expression of oral stereotypies when housed indoors was predicted by the count of intervals feeding/ foraging (χ^2^ = 323.353, df = 1, *p *< 0.001) and ruminating (χ^2^ = 169.276, df = 1, *p *< 0.001). Feeding/ foraging (β = -0.327, SE = 0.018), and ruminating (β = -0.403, SE = 0.031) were both negatively related to oral stereotypies. Oral stereotypies were also significantly predicted by herd size (χ^2^ = 3.851, df = 1, *p *= 0.050), with more time spent performing oral stereotypies by giraffes living in larger herds (β = 0.146, SE = 0.074).

**Fig 9:**
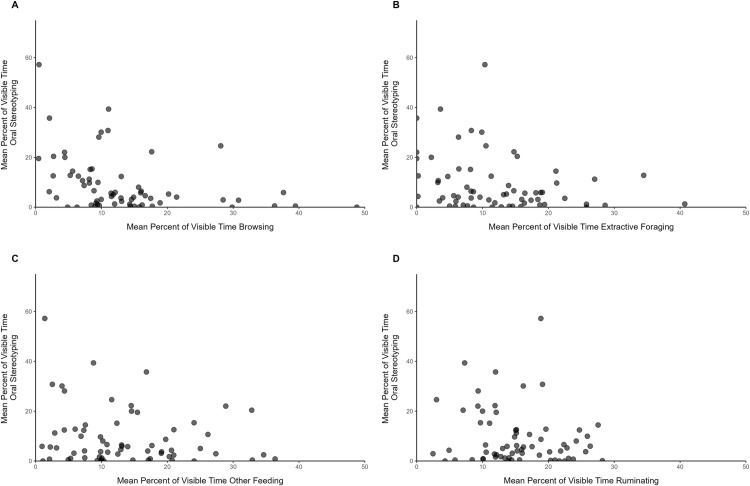
Scatterplots of each individual’s mean percent of time displaying oral stereotypies by their time spent displaying feeding-related behaviors.

The mean percent of time an individual giraffe spent exhibiting oral stereotypies was negatively correlated with their time spent browsing (r_s_ = -0.439, S = 68936, *p* < 0.001) and extractive foraging (r_s_ = -0.243, S = 59560, *p* = 0.049) but not with their time spent displaying other feeding behaviors (r_s_ = -0.123, S = 53801, *p* = 0.325) or ruminating (r_s_ = -0.111, S = 53233, *p* = 0.374; Fig 9).

### Inactive

**Fig 10:**
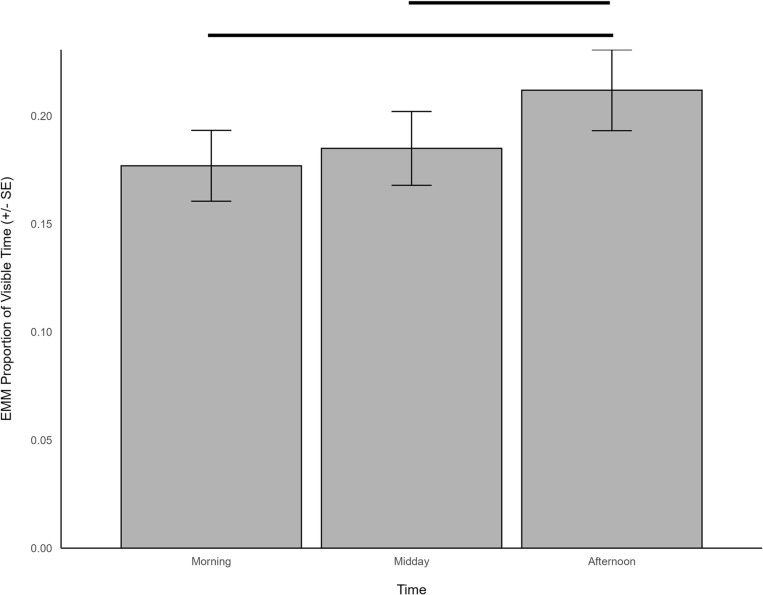
The estimated marginal means for significant predictors of inactive behavior when giraffes were housed outdoors. Significant differences are indicated with a line.

Inactive behavior when housed outdoors was significantly predicted by temperature (χ^2^ = 60.293, df = 1, *p *< 0.001) and time (χ^2^ = 26.558, df = 2, *p *< 0.001). Inactive behavior was observed to increase in the afternoon (EMM = 0.212, SE = 0.019) as compared to morning (EMM = 0.177, SE = 0.016; z = -5.029, p < 0.001) and midday (EMM = 0.185, SE = 0.017; z = -3.626, p < 0.001; Fig 10), and demonstrated a positive relationship with temperature (β = 0.218, SE = 0.028). In addition, a trend to significance was noted for outdoor habitat size (χ^2^ = 2.901, df = 1, *p *= 0.089), which was positively related to inactivity (β = 0.162, SE = 0.095), and whether the zoo offered public feeding (χ^2^ = 3.37, df = 1, *p *= 0.067), with increased inactive behavior observed at zoos with public feeding opportunities (EMM = 0.224, SE = 0.017) compared to zoos with no public feeding (EMM = 0.162, SE = 0.027).

When indoors, inactive behavior was significantly predicted by herd size (χ^2^ = 4.213, df = 1, *p *= 0.040), with less time spent inactive for giraffe living in larger herds (β = -0.039, SE = 0.019). A trend to significance was noted for time of day (χ^2^ = 5.224, df = 2, *p *= 0.073), with inactivity higher in the morning (EMM = 0.157, SE = 0.014) compared to midday (0.131, SE = 0.014; z = 2.268, *p* = 0.060).

### Locomotion

**Fig 11:**
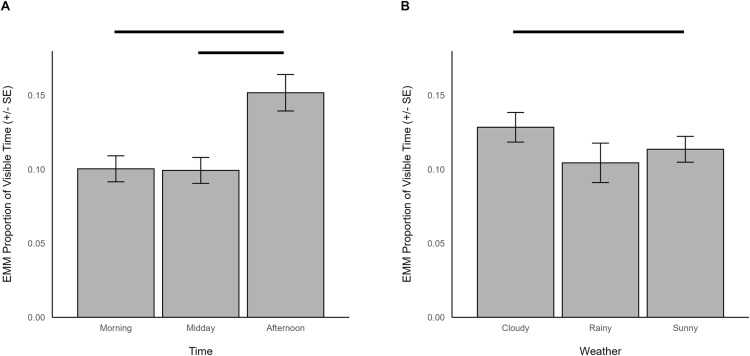
The estimated marginal means for significant predictors of locomotion behavior when giraffes were housed outdoors. Significant differences are indicated with a line.

Locomotion when housed outdoors was significantly predicted by time (χ^2^ = 149.558, df = 2, *p *< 0.001), temperature (χ^2^ = 75.553, df = 1, *p *< 0.001), herd size (χ^2^ = 3.911, df = 1, *p *= 0.048), and weather (χ^2^ = 15.841, df = 2, *p *< 0.001). Specifically, locomotion was more frequent in the afternoon (EMM = 0.152, SE = 0.012) compared to morning (EMM = 0.100, SE = 0.009; z = -10.789, p < 0.001) or midday (EMM = 0.099, SE = 0.009; z = 10.464, p < 0.001; Fig 11A), was negatively related to temperature (β = -0.237, SE = 0.027) and herd size (β = -0.035, SE = 0.018), and occurred more when cloudy (EMM = 0.128, SE = 0.010) than sunny (EMM = 0.114, SE = 0.009; z = 3.768, p < 0.001; Fig 11B).

**Fig 12:**
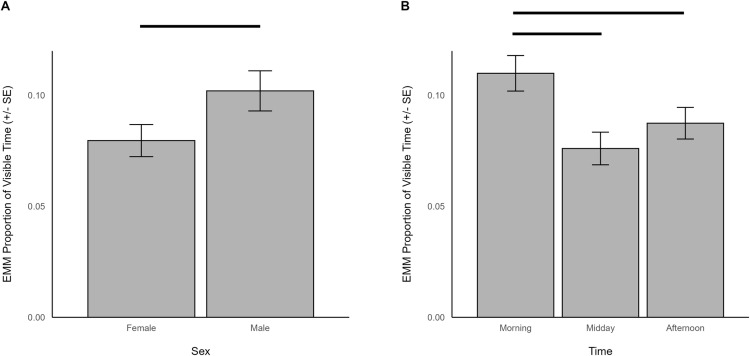
The estimated marginal means for significant predictors of locomotion behavior when giraffes were housed indoors. Significant differences are indicated with a line.

When indoors, locomotion was predicted by sex (χ^2^ = 4.208, df = 2, *p *= 0.040), time (χ^2^ = 19.331, df = 2, *p *< 0.001), and herd size (χ^2^ = 7.804, df = 1, *p *= 0.005). Specifically, there was more locomotion in males (EMM = 0.102, SE = 0.009) than females (EMM = 0.080, SE = 0.007; Fig 12A). Giraffes also spent more time locomoting in the morning (EMM = 0.110, SE = 0.008) than during midday (EMM = 0.076, SE = 0.007; z = 4.083, p < 0.001) or afternoon (EMM = 0.087, SE = 0.007; z = 2.935, p = 0.009; Fig 12B). Giraffes living in larger herds spent less time locomoting (β = -0.040, SE = 0.014).

## Discussion

To continually improve care for giraffes in zoos, a better understanding of the factors influencing behavior in managed settings is needed. In this study, we expand upon previous work describing the behavior patterns of giraffes housed across AZA-accredited zoos [7] to evaluate select organizational, environmental, temporal, and individual factors on giraffes’ behavior. Giraffe behavior differed between indoor and outdoor housing conditions in several ways. Across housing conditions, time of day had a greater impact on giraffe behavior than organizational or individual characteristics. When outdoors, temperature also strongly influenced giraffe behavior. From a broad conceptual standpoint, this may reflect the adaptive purpose of behavior, as animals adjust their behavior in response to dynamic changes in their environment.

One finding that was clear and reinforced previous work is that the expression of oral stereotypies was strongly associated with specific types of feeding behavior, specifically browsing and foraging. As animals showed less browsing and extractive foraging behavior which involves greater tongue manipulation and smaller quantities of food per attempt, they showed more oral stereotypic behavior. This was not the case for other feeding behaviors which, by definition, involved less processing. This relationship reinforces the importance of these specific, more demanding foraging behaviors for mitigating stereotypic expression. Further, habitat size, herd size, and the presence of public feeding programs, which are static characteristics of an organization, were predictive of browsing behavior. We consider each factor in detail below and interpret these findings in light of past research.

### Indoor and outdoor housing influences on behavior

Giraffes in this study spent more time browsing, extractive foraging, and displaying oral stereotypies when indoors and less time engaged in other feeding behaviors, inactive, and locomoting. Overall, these differences were minimal, with most behaviors having an estimated difference of five percent of their visible time or less between housing conditions. For most zoos in this study, housing changed seasonally, with increased time spent indoors during winter months. Giraffes have been previously reported to spend more time feeding in winter, when housed indoors [12,14]. Although there are many differences between indoor and outdoor housing, one possible factor influencing feeding behaviors may be related to how food was provisioned. Many zoos in this study offered public giraffe feeding opportunities. These programs are conducted outdoors and, as discussed below, were associated with a shift in feeding behaviors, with less time browsing and more time spent in other feeding behaviors. Thus, increased browsing when housed indoors may reflect the lack of public feeding opportunities. In addition, seasonal changes in diet are possible [37]. As Razal et al. [14] noted, giraffes in winter are more likely to have increased non-leafy browse which may require additional tongue manipulation, thereby extending feeding bouts. Increased feeding in the dry season has also been reported in wild giraffes and attributed to decreased food quality [16]. Dietary differences between seasons and thus, housing conditions, are likely but were not assessed directly in this study.

Activity changes in this study were also similar to that of Razal et al. [14], with more locomotion observed when giraffes were housed outdoors. The authors speculated this difference may reflect the larger size of outdoor habitats, with feeding and enrichment being dispersed over a greater area. Although this explanation is plausible, we did not observe increased locomotion in larger outdoor or indoor habitats (discussed below). If this difference is related to habitat size, this suggests that increased locomotion when outdoors compared to indoors may relate to the large magnitude of difference between indoor and outdoor habitat sizes. Differences in social density across indoor and outdoor habitats may have also played a role. Giraffes in the current study were also more inactive when outdoors, which may reflect decreased social pressure resulting from increased available space. These broad changes in activity levels may reflect an overall shift in activity budgets, from a more concentrated use of space for feeding when indoors to a more diverse and distributed behavior profile when outdoors. Although oral stereotypic behavior was observed more often indoors, contrary to the findings of Razal et al. [14], this behavioral difference was minimal (0.4% difference in visible time) and likely did not represent a biological difference.

### Organizational influences on behavior

#### Habitat size.

It has been suggested that larger habitats may promote increased activity [38]. Several studies on different species have found support for this (e.g., elephants, [39]; penguins, [40]). In this study, outdoor habitat sizes differed drastically across organizations, with the largest outdoor habitat (263,043 m^2^) being 566 times bigger than the smallest (464 m^2^). Indoor habitats were generally much smaller and more similarly sized between organizations (median size was 268 m^2^). Across zoos, we observed few behavioral differences based on habitat size.

Notably, we did not see increased locomotion in larger habitats. As others have noted, habitat complexity may be more important than size for some behaviors [41]. In a study at the Dallas Zoo where elephants were moved between different habitats that controlled for size and complexity, Scott and Ladue [41] found increased activity and foraging by elephants moved to more complex habitats, regardless of their size. Indeed, studies that have found increased activity in larger habitats have often compared behavior in older, smaller habitats with less complexity to newer, larger habitats with more complexity [39,40], making it unclear if size or complexity ultimately influenced behavior. Although habitat complexity was not assessed in this study, it is possible that larger habitats may have had lower overall complexity given the logistical constraints of servicing larger spaces. This may also potentially explain the negative relationship between habitat size and browsing behavior that was observed in outdoor housing conditions, as there may have been greater challenges for animal keepers in placing browse in larger habitats. A similar (negative) relationship was also noted between indoor habitat size and extractive foraging behaviors. Interestingly, giraffes spent more time engaged in other feeding behaviors in larger outdoor and indoor habitats. These other feeding behaviors represent times when food was provided in troughs or other easily accessible places and, consequently, would require less servicing time for staff to set up. More work is needed to evaluate potential differences in food provisioning and overall complexity based on habitat size. Recently, a framework for evaluating habitats has been proposed that assesses habitats along multiple broad components of design [42]. This framework may serve as a valuable starting point in future multi-institutional studies for describing habitats in standardized and measurable ways.

It should also be noted that outdoor and indoor habitat sizes in this study were analyzed based on the total space at a zoo and may not reflect the actual amount of space available to an individual, as habitats may be subdivided into multiple stalls or paddocks. This limitation may have resulted in the minimal behavioral differences observed based on habitat size and a more detailed examination of space (e.g., space experience metric, [5]) could reveal greater insights. In addition, it may be valuable to consider the detailed space use of animals in relation to the distribution of resources (e.g., feeding opportunities) throughout spaces, along with the density of animals within a space, which may better reflect the individual experience.

#### Herd size.

In the wild, the size of giraffe herds has been found to change in response to local resource availability through complex fission-fusion social structures [43,44]. In zoos, however, herd size and composition are typically fixed. In this study, only one zoo reported a fission-fusion management style for their giraffe. With a fixed composition, herd size might be expected to influence behavior as individuals in larger herds could have increased competition for food and increased, and potentially less avoidable, social interactions [45]. Alternatively, giraffes may feel “safer” in larger herds [46].

Overall, herd size influenced multiple giraffe behaviors, particularly when housed indoors. Under outdoor and indoor conditions, herd size was found to negatively influence locomotion. One interpretation is that this might indicate lowered vigilance and suggest a positive response, as one recent study found no overall effect of herd size on resting or activity in wild giraffes but did observe giraffes spent more time resting when in closer proximity to conspecifics [46]. Although social proximity was not evaluated in the current study, herd size and social proximity are likely to be more closely associated in zoos, given space constraints. However, when housed indoors, herd size was also associated with less time spent inactive, suggesting this difference is unlikely to represent decreased vigilance through a “dilution effect”.

Alternatively, decreased locomotion may reflect overcrowding, with less space to move per individual in larger herds. In their study, Hejcmanová et al. [46] reported giraffes in the wild had a mean distance of 57.7 m to other giraffes. Here, the median outdoor space available per individual was 766 m^2^ which, assuming a circular “bubble” of space, would only provide a maximum distance of 15.6 m between giraffes if they were evenly dispersed through the space, an unrealistic scenario. In addition, giraffes in zoos are often housed with other species (14/18 zoos in this study), which may place additional constraints on the available space. The potential for overcrowding is more acute in indoor habitats, where giraffes in this study had a median available space per individual of 61 m^2^, with a maximum distance of only 4.4 m possible between giraffes if evenly dispersed. Giraffes in larger herds spent more time displaying oral stereotypies when indoors, a potential response to increased social pressure. Although social behaviors were not assessed here given their rarity (<2% of their visible time budget; [7]), a recent study found increased aggression between two female giraffes when housed in a smaller outdoor habitat [45]. In their study, the available space per individual in the small outdoor habitat (120 m^2^) was much smaller than individuals experienced in outdoor habitats in the present study but approximately twice the size of indoor areas. Overall, these results suggest giraffe habitats for large herds may need to be bigger than previously recognized. More work is needed to explore the impact of social density on giraffe behavior.

Herd size was also observed to influence feeding behaviors when housed indoors, with less time browsing and more time extractive foraging for giraffes in larger herds. This shift in feeding may reflect diet availability, as these zoos were primarily in northern climates where access to large quantities of browse needed for large herds would be more challenging to procure, particularly during winter months when giraffes are more commonly housed indoors [14]. In evaluating the design of a browse plantation, Höllerl et al. [47] estimated that one hectare was needed to feed three giraffes. For the largest herd in this study, this would require 5 hectares of space to provide consistent, year-round access to browse.

#### Public feeding.

Public feeding opportunities for giraffes are common in zoos, with 14 of the 18 zoos participating in this study offering this experience. Public feeding is attractive for zoos, as these programs offer unique guest experiences and can generate revenue. However, it is important to consider the potential implications these programs may have on animal behavior and, consequently, wellbeing. Orban et al. [11] compared giraffe behavior between six zoos with public feeding to three zoos without these experiences. They found giraffes in public feeding programs may be more inactive, particularly in open programs that occur throughout the day. Giraffes were also observed to ruminate less at zoos with public feeding. In the current study, we similarly identified a trend of increased time inactive at zoos with public feeding. As Orban et al. [11] suggested, this may reflect time the giraffes spend waiting for these interactions. In addition, we observed a shift in feeding behaviors. At zoos with public feeding, less time was spent browsing and more time was spent displaying other feeding behaviors, a behavioral grouping that included those feeding behaviors requiring minimal oral manipulation. This difference in feeding behaviors likely reflects the food available to giraffes in public feeding programs, which typically include lettuce and other vegetables that are easy to quickly hand feed but are unlikely to include browse that requires more oral processing by the giraffes. This limitation may have consequences on the display of oral stereotypies, as those stereotypic behaviors were observed to be negatively correlated with time spent browsing and extractive foraging but not time spent in other feeding behaviors (discussed further below).

### Environmental influences on behavior

Giraffes have been shown to have a suite of physiological and behavioral adaptations to minimize heat load [20,48,49]. Thermoregulation has even been suggested to explain, in part, the iconic shape of giraffes, with their tall but compact body minimizing the exposure to solar radiation and maximizing the surface area of the neck and legs for heat transfer [48]. In this study, environmental factors played a large role in giraffe behavior. Changes in general activity in response to temperature, with increased inactivity and decreased movement under hotter temperatures, are consistent with past research in giraffes [15,50] and suggest a behavioral thermoregulation strategy. Changes in feeding behaviors are harder to interpret. Decreased extractive foraging and ruminating may be indicative of a thermoregulatory response [51]. However, the positive relationship between temperature and other feeding behavior is less clear. Although not evaluated in the current study, shade use may be another key behavioral thermoregulation strategy of giraffe [18]. This may explain the increased locomotion observed in this study when cloudy. Overall, these findings demonstrate that temperature can have a strong influence on giraffe activity and encourage additional studies on the influence of shade availability on giraffe behavior.

Oral stereotypic behaviors occurred more frequently in colder temperatures in this study and a similar response has been reported by others [52]. Given their adaptations for living in savannah environments, giraffes are consequentially poorly suited for cold temperatures, which may be a source of stress for individuals living in northern zoos [53]. Elevated stereotypic behavior under colder temperatures has been reported in other species [54,55] and may indicate a stress response. The United States Department of Agriculture [56] recommends giraffes have access to heated areas when temperatures are below 10 C. In this study, 167 sessions were recorded when giraffes were housed outdoors below 10 C, representing 2.5% of the outdoor dataset. Although this was rare overall and it is unclear how long giraffe were housed in these conditions, this does suggest zoos may need to more closely monitor outdoor temperatures and consider providing indoor access at higher temperatures to ensure individuals have the choice to avoid cold conditions.

### Temporal influences on behavior

As expected, giraffes in this study showed a number of behavioral changes throughout the day. When outdoors, they spent more time engaged in feeding behaviors and less time ruminating during the morning hours. This was followed by increased inactivity and locomotion in the afternoon. This is broadly similar with what has been reported in wild giraffes, with feeding peaks in the morning and early evening and a trough of inactivity in the middle of the day [16,18,20]. Although we did not observe a secondary peak in feeding, this may reflect the daytime observation window of the current study, as another study that considered giraffe behavior over a 24-hr period at two zoos did detect a secondary peak occurring at 1900 hours [21]. This biphasic activity pattern has been suggested to be a thermoregulatory response to diurnal temperatures [16]. Although cooler temperatures in the afternoon may have contributed to increased locomotion during this time, this may also be a sign of anticipatory behavior.

When outdoors, oral stereotypies were most common in the middle of the day. As feeding peaked in the morning, it is plausible there was less food available during midday and increased oral stereotypies at this time may represent frustration. Frustrated foraging behavior has been suggested to be an underlying cause of oral stereotypies in ungulates [57]. This is supported by the (negative) relationship between foraging behavior and oral stereotypies (discussed further below). Alternatively, it is also possible increased oral stereotypies during midday may reflect boredom more than frustration. If this was the case, this might shift the interpretation of oral stereotypies from negative to neutral, from an affective standpoint. This suggestion of boredom may be partially supported by the observation of these behaviors in the wild [8]. Ultimately, more work is needed to unravel the motivational basis of oral stereotypies in giraffes.

Giraffe behavior when housed indoors appeared to be less influenced by time of day. As these areas are temperature-controlled, this may reflect a lower environmental pressure on behavior. When indoors, giraffes spent less time browsing and more time inactive and locomoting in the morning compared to other times of day. This pattern is opposite of what was observed when giraffes were housed outdoors. Although not recorded in this study, it is possible this may reflect differences in husbandry scheduling when giraffes are housed indoors compared to outdoors. Future studies should consider tracking the timing of husbandry events alongside behavior to tease apart in more detail the immediate effects of husbandry on behavioral responses.

### Individual influences on behavior

Differences in sex and age were noted and represent a contrast with what has been reported for giraffes living in the wild. In this study, males were observed to spend more time browsing and extractive foraging and less time ruminating than females, when housed outdoors. In addition, subadults spent more time browsing when indoors and a trend when outdoors. This is opposite to what has been reported in the wild [15–18]. It has been suggested that feeding time budgets in the wild may represent a trade off with vigilance, with males and subadults spending less time feeding to allow more time for vigilance [17]. In zoos, a lack of predation risks and need for vigilance might be expected to shift this dynamic. Food availability may also play a role, as male giraffes in the wild have been suggested to meet their caloric needs with a reduced time budget by feeding faster and spending longer ruminating. It is possible that male giraffes in zoos have less food available to them at one time than in the wild, limiting their ability to fill their gut when they feed.

In line with past studies, oral stereotypic behaviors were found to be negatively related with feeding behaviors [9,11,21]. In behavior models controlling for other factors, this effect was significant across both outdoor and indoor environments. In addition, when evaluating these behaviors directly against each other, differences were noted between feeding-related behaviors. Individuals that spent more time browsing or extractive foraging were observed to spend less time displaying oral stereotypies but no difference was found based on the time an individual spent in other feeding behaviors or ruminating. This difference between feeding behaviors is intriguing, as browsing and extractive foraging behaviors represent the natural feeding behavior of giraffes. Promoting natural feeding behaviors has been a focus of zoos and a recent report comparing estimates from this study to past research suggested community-wide progress in these efforts [7]. The relationship between oral stereotypies and feeding behaviors observed in this study further reinforces the need for continued efforts to promote browsing and extractive foraging behaviors for giraffes living in zoos.

## Conclusion

**Fig 13:**
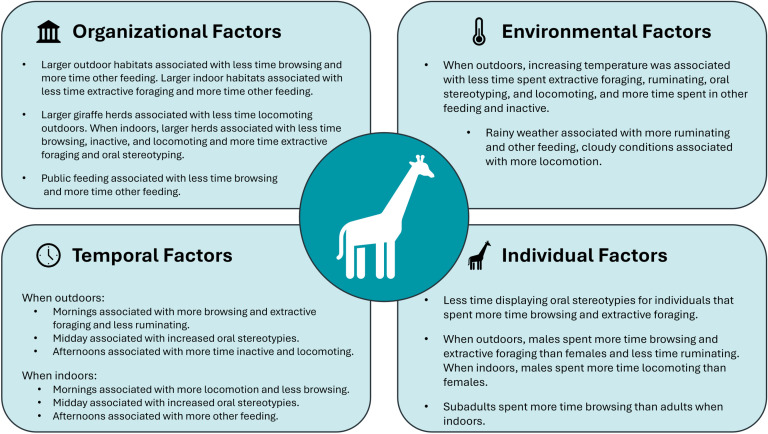
Summary of factors influencing giraffe behavior in this study.

Large, multi-institutional studies are instrumental in uncovering the factors influencing animal behavior in zoos and aquariums. This study highlights a complex array of influences on the behavior of giraffes housed in AZA-accredited zoos (Fig 13). Several findings have direct implications for giraffe care and wellbeing. For example, oral stereotypies – a common and concerning behavior in giraffes – were negatively correlated with time spent browsing and extractive foraging. A negative relationship between oral stereotypies and feeding has been documented by others but these findings suggest an important role for natural foraging behaviors. To mitigate oral stereotypies, zoos should prioritize browsing and extractive foraging opportunities for giraffes. Giraffes living at zoos with public feeding opportunities spent less time browsing, more time in other feeding behaviors, and showed a trend of increased inactivity, suggesting these programs may negatively influence giraffe behavior. This study failed to reveal a significant relationship between habitat size and locomotion, challenging past assumptions that larger habitats encourage greater activity. This finding suggests other factors, such as habitat complexity or daily feeding and enrichment routines, may have a more substantial impact on giraffe activity but this remains to be tested. Overall, these findings provide a foundation for additional research to develop evidence-based husbandry practices that enhance the wellbeing of giraffes in managed care.

## Supporting information

S1 Dataset Raw data. (XLSX)

S2 Table Ethogram. (DOCX)

S3 Table Summary count of sessions recorded per zoo by housing condition and time of day (morning: < 11:30 AM; midday: 11:30 AM to 1:30 PM; afternoon: > 1:30 PM). (DOCX)

S4 Table Statistical output of behavior models. (DOCX)
