# Supplementary material for: Factors shaping giraffe behavior in U.S. zoos: A multi-institutional study to inform management
Source: PLoS One. 2025 May 29;20(5):e0324248. doi: 10.1371/journal.pone.0324248 (PMC12121815; doi:10.1371/journal.pone.0324248)
Supplement: S2 Table — (DOCX) [file pone.0324248.s002.docx]

**S2 Table. Ethogram.**

| **Behavior Name** | **Behavior Category** | **Definition** |
| --- | --- | --- |
| Standing | Inactive | Animal is upright with weight supported on feet and not performing another behavior listed. |
| Sitting | Inactive | Animal has weight supported on legs or ventral surface (c.f., “rest (lying)” [25]). May be alert with head elevated or sleeping with head resting on their body. |
| Browsing/ Feed | Feed/ Forage/ Drink | Animal is using tongue or mouth to strip or pluck leaves or bark from a branch (can include environmental foliage as well as diet items). This includes chewing and consumption of food items gained through browsing. |
| Extractive Foraging/ Feed | Feed/ Forage/ Drink | Animal is using tongue or mouth to extract food from within an enclosed object (e.g., hanging extractive feeding bags or buckets). This includes chewing and consumption of food items gained through extractive foraging. |
| Ruminating | Feed/ Forage/ Drink | Regurgitation and chewing cud of previously eaten food. Does not include periods of chewing which might accompany foraging and should be recorded as “Feeding.” |
| Other Feeding/ Drinking | Feed/ Forage/ Drink | Animal is performing any other feeding behavior (e.g., feeding from troughs, grazing on grass, foraging across substrate, guest hand feeding). |
| Locomotion | Locomotion | Animal is moving at least one body’s length in a non-stereotypical manner. |
| Tongue Play | Stereotypy | Animal is moving tongue outside of mouth in a repetitive, twisting or rolling movement. May have food item present but not actively chewing food. |
| Repetitive Licking | Stereotypy | Animal is repeatedly moving tongue across a non-food, stationary object (e.g., walls, fencing, or trees). |
| Pacing | Stereotypy | Animal is walking in a repetitive manner along a fixed path without an apparent goal or function. The animal must move along the path three times to qualify as pacing. [Note: If an interval occurs during the first two transects and the animal continues into a pacing bout, score pacing]. |
| Other Stereotypy | Stereotypy | Animal is performing any other non-functional, invariant, and repetitive behavior not listed above (please score whether the stereotypy type is Oral, Motor, Locomotor, or Other). |
| Other Solitary | Other Solitary | Animal is performing any other solitary behavior, including but not limited to self-maintenance behaviors, exploratory behaviors, and elimination behaviors. |
| Affiliative | Social | Animal makes physical contact with another conspecific individual in an affiliative manner, including rubbing necks, heads, bodies, or muzzles or sniffing and licking the muzzle or non-anogenital area of the body. |
| Sexual | Social | Animal is physically mounting or attempting to mount a conspecific animal or investigating the animal or environment in a sexual manner (e.g., anogenital exam, urine investigation, flehmen). |
| Agonistic | Social | Animal performs any aggressive behavior, either with or without contact, or any displacement/ avoidance behavior. |
| Other Social Behavior | Social | Animal is performing a social behavior not previously listed. |
| Behavior Obscured | Not Visible | The behavior of the animal cannot be determined but the location of the animal is known and in the habitat spaces under observation (i.e., record a corresponding space use location). |
| Animal Not Visible | Not Visible | The animal is completely not visible and its location is unknown (i.e., do not record a space use location) or in an off-exhibit area not under observation. |
