# Supplementary material for: Factors shaping giraffe behavior in U.S. zoos: A multi-institutional study to inform management
Source: PLoS One. 2025 May 29;20(5):e0324248. doi: 10.1371/journal.pone.0324248 (PMC12121815; doi:10.1371/journal.pone.0324248)
Supplement: S3 Table — (DOCX) [file pone.0324248.s003.docx]

**S3 Table. Summary count of sessions recorded per zoo by housing condition and time of day (morning: <11:30 AM; midday: 11:30 AM to 1:30 PM; afternoon: >1:30 PM).**

|  |  | Outdoor Sessions | | | Indoor Sessions | | |
| --- | --- | --- | --- | --- | --- | --- | --- |
| Zoo ID | Total Sessions | Morning | Midday | Afternoon | Morning | Midday | Afternoon |
| Zoo 1 | 1381 | 422 | 599 | 19 | 132 | 161 | 48 |
| Zoo 2 | 450 | 98 | 106 | 56 | 105 | 54 | 31 |
| Zoo 3 | 734 | 153 | 90 | 139 | 177 | 71 | 104 |
| Zoo 4 | 127 | 3 | 64 | 60 | 0 | 0 | 0 |
| Zoo 5 | 420 | 23 | 51 | 85 | 80 | 47 | 134 |
| Zoo 6 | 372 | 147 | 62 | 163 | 0 | 0 | 0 |
| Zoo 7 | 295 | 23 | 15 | 49 | 33 | 29 | 146 |
| Zoo 8 | 506 | 210 | 108 | 188 | 0 | 0 | 0 |
| Zoo 9 | 576 | 265 | 179 | 132 | 0 | 0 | 0 |
| Zoo 10 | 512 | 201 | 108 | 193 | 3 | 0 | 7 |
| Zoo 11 | 18 | 4 | 10 | 4 | 0 | 0 | 0 |
| Zoo 12 | 356 | 144 | 98 | 114 | 0 | 0 | 0 |
| Zoo 13 | 307 | 109 | 43 | 70 | 39 | 15 | 31 |
| Zoo 14 | 841 | 379 | 226 | 236 | 0 | 0 | 0 |
| Zoo 15 | 334 | 46 | 52 | 136 | 33 | 7 | 60 |
| Zoo 16 | 340 | 112 | 91 | 137 | 0 | 0 | 0 |
| Zoo 17 | 523 | 119 | 44 | 109 | 142 | 38 | 71 |
| Zoo 18 | 238 | 24 | 117 | 72 | 3 | 1 | 21 |
