# Supplementary material for: Factors shaping giraffe behavior in U.S. zoos: A multi-institutional study to inform management
Source: PLoS One. 2025 May 29;20(5):e0324248. doi: 10.1371/journal.pone.0324248 (PMC12121815; doi:10.1371/journal.pone.0324248)
Supplement: S4 Table — (DOCX) [file pone.0324248.s004.docx]

**S4 Table. Statistical output of behavior models.**

| Behavior | Habitat | Fixed Effects | Estimate | S.E. | Z | *p* | χ^2^ | df | *p* |
| --- | --- | --- | --- | --- | --- | --- | --- | --- | --- |
| Browsing | Outdoor | Intercept | -1.364 | 0.300 | -4.551 | <0.001 |  |  |  |
|  |  | Age Class (Subadult) | 0.300 | 0.165 | 1.817 | 0.069 | 3.302 | 1 | 0.069 |
|  |  | Sex (Male) | 0.483 | 0.156 | 3.103 | 0.002 | 9.631 | 1 | 0.002 |
|  |  | Time Category (Midday) | -0.216 | 0.062 | -3.499 | <0.001 | 32.953 | 2 | <0.001 |
|  |  | Time Category (Afternoon) | -0.370 | 0.067 | -5.559 | <0.001 |  |  |  |
|  |  | Temperature | 0.021 | 0.040 | 0.533 | 0.594 | 0.284 | 1 | 0.594 |
|  |  | Herd Size | 0.037 | 0.038 | 0.972 | 0.331 | 0.946 | 1 | 0.331 |
|  |  | Habitat Size | -0.643 | 0.154 | -4.178 | <0.001 | 17.46 | 1 | <0.001 |
|  |  | Weather (Rainy) | 0.177 | 0.154 | 1.153 | 0.249 | 5.246 | 2 | 0.073 |
|  |  | Weather (Sunny) | -0.092 | 0.055 | -1.670 | 0.095 |  |  |  |
|  |  | Public Feeding (Yes) | -1.250 | 0.350 | -3.571 | <0.001 | 12.754 | 1 | <0.001 |
| Browsing | Indoor | Intercept | -1.611 | 0.238 | -6.766 | <0.001 |  |  |  |
|  |  | Age Class (Subadult) | 0.448 | 0.223 | 2.013 | 0.044 | 4.054 | 1 | 0.044 |
|  |  | Sex (Male) | 0.112 | 0.211 | 0.531 | 0.596 | 0.282 | 1 | 0.596 |
|  |  | Time of Day (Midday) | 0.439 | 0.114 | 3.848 | <0.001 | 17.522 | 2 | <0.001 |
|  |  | Time of Day (Afternoon) | 0.344 | 0.109 | 3.147 | 0.002 |  |  |  |
|  |  | Herd Size | -0.060 | 0.030 | -2.005 | 0.045 | 4.02 | 1 | 0.045 |
|  |  | Habitat Size | -0.128 | 0.143 | -0.891 | 0.373 | 0.793 | 1 | 0.373 |
| Extractive Foraging | Outdoor | Intercept | -1.751 | 0.555 | -3.153 | 0.002 |  |  |  |
|  |  | Age Class (Subadult) | -0.094 | 0.176 | -0.530 | 0.596 | 0.281 | 1 | 0.596 |
|  |  | Sex (Male) | 0.449 | 0.167 | 2.679 | 0.007 | 7.177 | 1 | 0.007 |
|  |  | Time Category (Midday) | -0.161 | 0.069 | -2.352 | 0.019 | 13.352 | 2 | 0.001 |
|  |  | Time Category (Afternoon) | -0.255 | 0.073 | -3.478 | <0.001 |  |  |  |
|  |  | Temperature | -0.258 | 0.045 | -5.739 | <0.001 | 32.934 | 1 | <0.001 |
|  |  | Herd Size | -0.010 | 0.073 | -0.140 | 0.888 | 0.02 | 1 | 0.888 |
|  |  | Habitat Size | -0.126 | 0.286 | -0.439 | 0.661 | 0.193 | 1 | 0.661 |
|  |  | Weather (Rainy) | -0.115 | 0.190 | -0.605 | 0.545 | 0.962 | 2 | 0.618 |
|  |  | Weather (Sunny) | 0.039 | 0.062 | 0.624 | 0.533 |  |  |  |
|  |  | Public Feeding (Yes) | -0.908 | 0.641 | -1.416 | 0.157 | 2.004 | 1 | 0.157 |
|  | Indoor | Intercept | -2.086 | 0.308 | -6.770 | <0.001 |  |  |  |
|  |  | Age Class (Subadult) | 0.181 | 0.199 | 0.910 | 0.363 | 0.827 | 1 | 0.363 |
|  |  | Sex (Male) | -0.083 | 0.187 | -0.443 | 0.658 | 0.196 | 1 | 0.658 |
|  |  | Time of Day (Midday) | 0.211 | 0.120 | 1.764 | 0.078 | 3.148 | 2 | 0.207 |
|  |  | Time of Day (Afternoon) | 0.097 | 0.111 | 0.871 | 0.384 |  |  |  |
|  |  | Herd Size | 0.094 | 0.043 | 2.179 | 0.029 | 4.747 | 1 | 0.029 |
|  |  | Habitat Size | -0.564 | 0.200 | -2.824 | 0.005 | 7.978 | 1 | 0.005 |
| Other Feeding | Outdoor | Intercept | -3.151 | 0.283 | -11.123 | <0.001 |  |  |  |
|  |  | Age Class (Subadult) | 0.177 | 0.151 | 1.170 | 0.242 | 1.37 | 1 | 0.242 |
|  |  | Sex (Male) | 0.051 | 0.144 | 0.352 | 0.725 | 0.124 | 1 | 0.725 |
|  |  | Time Category (Midday) | 0.101 | 0.056 | 1.790 | 0.073 | 36.184 | 2 | <0.001 |
|  |  | Time Category (Afternoon) | -0.285 | 0.063 | -4.518 | <0.001 |  |  |  |
|  |  | Temperature | 0.113 | 0.037 | 3.079 | 0.002 | 9.479 | 1 | 0.002 |
|  |  | Herd Size | -0.058 | 0.033 | -1.770 | 0.077 | 3.134 | 1 | 0.077 |
|  |  | Habitat Size | 0.303 | 0.142 | 2.135 | 0.033 | 4.559 | 1 | 0.033 |
|  |  | Weather (Rainy) | -0.481 | 0.172 | -2.790 | 0.005 | 9.504 | 2 | 0.009 |
|  |  | Weather (Sunny) | 0.039 | 0.051 | 0.773 | 0.439 |  |  |  |
|  |  | Public Feeding (Yes) | 1.789 | 0.336 | 5.318 | <0.001 | 28.28 | 1 | <0.001 |
|  | Indoor | Intercept | -2.087 | 0.301 | -6.942 | <0.001 |  |  |  |
|  |  | Age Class (Subadult) | 0.209 | 0.112 | 1.871 | 0.061 | 3.501 | 1 | 0.061 |
|  |  | Sex (Male) | 0.041 | 0.114 | 0.362 | 0.718 | 0.131 | 1 | 0.718 |
|  |  | Time of Day (Midday) | 0.016 | 0.124 | 0.130 | 0.897 | 6.752 | 2 | 0.034 |
|  |  | Time of Day (Afternoon) | 0.259 | 0.106 | 2.434 | 0.015 |  |  |  |
|  |  | Herd Size | -0.072 | 0.044 | -1.660 | 0.097 | 2.756 | 1 | 0.097 |
|  |  | Habitat Size | 0.466 | 0.190 | 2.451 | 0.014 | 6.009 | 1 | 0.014 |
| Ruminating | Outdoor | Intercept | -1.520 | 0.284 | -5.359 | <0.001 |  |  |  |
|  |  | Age Class (Subadult) | -0.210 | 0.123 | -1.708 | 0.088 | 2.918 | 1 | 0.088 |
|  |  | Sex (Male) | -0.266 | 0.118 | -2.243 | 0.025 | 5.031 | 1 | 0.025 |
|  |  | Time Category (Midday) | 0.144 | 0.061 | 2.356 | 0.018 | 6.189 | 2 | 0.045 |
|  |  | Time Category (Afternoon) | 0.109 | 0.061 | 1.791 | 0.073 |  |  |  |
|  |  | Temperature | -0.100 | 0.037 | -2.668 | 0.008 | 7.117 | 1 | 0.008 |
|  |  | Herd Size | -0.002 | 0.036 | -0.055 | 0.956 | 0.003 | 1 | 0.956 |
|  |  | Habitat Size | -0.099 | 0.145 | -0.684 | 0.494 | 0.467 | 1 | 0.494 |
|  |  | Weather (Rainy) | 0.415 | 0.154 | 2.695 | 0.007 | 7.5 | 2 | 0.024 |
|  |  | Weather (Sunny) | 0.059 | 0.054 | 1.101 | 0.271 |  |  |  |
|  |  | Public Feeding (Yes) | -0.292 | 0.327 | -0.893 | 0.372 | 0.797 | 1 | 0.372 |
|  | Indoor | Intercept | -2.043 | 0.326 | -6.257 | <0.001 |  |  |  |
|  |  | Age Class (Subadult) | -0.183 | 0.173 | -1.059 | 0.290 | 1.121 | 1 | 0.29 |
|  |  | Sex (Male) | -0.267 | 0.161 | -1.658 | 0.097 | 2.749 | 1 | 0.097 |
|  |  | Time of Day (Midday) | -0.027 | 0.121 | -0.223 | 0.823 | 2.669 | 2 | 0.263 |
|  |  | Time of Day (Afternoon) | -0.175 | 0.110 | -1.594 | 0.111 |  |  |  |
|  |  | Herd Size | 0.017 | 0.045 | 0.379 | 0.705 | 0.143 | 1 | 0.705 |
|  |  | Habitat Size | 0.282 | 0.204 | 1.382 | 0.167 | 1.911 | 1 | 0.167 |
| Oral Stereotypies | Outdoor | Intercept | -1.592 | 0.576 | -2.764 | 0.006 |  |  |  |
|  |  | Feeding/Foraging/Drinking Count | -0.387 | 0.013 | -29.053 | <0.001 | 844.086 | 1 | <0.001 |
|  |  | Ruminating Count | -0.362 | 0.018 | -19.926 | <0.001 | 397.056 | 1 | <0.001 |
|  |  | Age Class (Subadult) | -0.352 | 0.344 | -1.024 | 0.306 | 1.048 | 1 | 0.306 |
|  |  | Sex (Male) | -0.395 | 0.317 | -1.245 | 0.213 | 1.549 | 1 | 0.213 |
|  |  | Time Category (Midday) | 0.125 | 0.074 | 1.686 | 0.092 | 7.027 | 2 | 0.03 |
|  |  | Time Category (Afternoon) | -0.096 | 0.084 | -1.138 | 0.255 |  |  |  |
|  |  | Temperature | -0.170 | 0.048 | -3.545 | <0.001 | 12.57 | 1 | <0.001 |
|  |  | Herd Size | 0.002 | 0.070 | 0.026 | 0.979 | 0.001 | 1 | 0.979 |
|  |  | Habitat Size | 0.469 | 0.304 | 1.545 | 0.122 | 2.388 | 1 | 0.122 |
|  |  | Weather (Rainy) | -0.354 | 0.208 | -1.703 | 0.089 | 2.901 | 2 | 0.234 |
|  |  | Weather (Sunny) | -0.020 | 0.066 | -0.307 | 0.759 |  |  |  |
|  |  | Public Feeding (Yes) | 0.241 | 0.687 | 0.350 | 0.726 | 0.123 | 1 | 0.726 |
|  | Indoor | Intercept | -2.296 | 0.535 | -4.293 | <0.001 |  |  |  |
|  |  | Feeding/Foraging/Drinking Count | -0.327 | 0.018 | -17.982 | <0.001 | 323.353 | 1 | <0.001 |
|  |  | Ruminating Count | -0.403 | 0.031 | -13.011 | <0.001 | 169.276 | 1 | <0.001 |
|  |  | Age Class (Subadult) | -0.145 | 0.273 | -0.533 | 0.594 | 0.285 | 1 | 0.594 |
|  |  | Sex (Male) | -0.309 | 0.255 | -1.212 | 0.226 | 1.469 | 1 | 0.225 |
|  |  | Time of Day (Midday) | 0.234 | 0.123 | 1.900 | 0.057 | 4.214 | 2 | 0.122 |
|  |  | Time of Day (Afternoon) | -0.016 | 0.128 | -0.129 | 0.897 |  |  |  |
|  |  | Herd Size | 0.146 | 0.074 | 1.962 | 0.050 | 3.851 | 1 | 0.05 |
|  |  | Habitat Size | 0.164 | 0.342 | 0.479 | 0.632 | 0.229 | 1 | 0.632 |
| Inactive | Outdoor | Intercept | -1.730 | 0.186 | -9.318 | <0.001 |  |  |  |
|  |  | Age Class (Subadult) | -0.014 | 0.121 | -0.119 | 0.905 | 0.014 | 1 | 0.905 |
|  |  | Sex (Male) | -0.055 | 0.111 | -0.494 | 0.621 | 0.244 | 1 | 0.621 |
|  |  | Time Category (Midday) | 0.054 | 0.044 | 1.229 | 0.219 | 26.558 | 2 | <0.001 |
|  |  | Time Category (Afternoon) | 0.224 | 0.045 | 5.029 | <0.001 |  |  |  |
|  |  | Temperature | 0.218 | 0.028 | 7.765 | <0.001 | 60.293 | 1 | <0.001 |
|  |  | Herd Size | -0.017 | 0.022 | -0.771 | 0.441 | 0.595 | 1 | 0.441 |
|  |  | Habitat Size | 0.162 | 0.095 | 1.703 | 0.089 | 2.901 | 1 | 0.089 |
|  |  | Weather (Rainy) | 0.060 | 0.118 | 0.508 | 0.611 | 0.329 | 2 | 0.848 |
|  |  | Weather (Sunny) | 0.014 | 0.038 | 0.366 | 0.715 |  |  |  |
|  |  | Public Feeding (Yes) | 0.404 | 0.220 | 1.835 | 0.067 | 3.366 | 1 | 0.067 |
|  | Indoor | Intercept | -1.659 | 0.179 | -9.270 | <0.001 |  |  |  |
|  |  | Age Class (Subadult) | -0.004 | 0.196 | -0.019 | 0.985 | 0 | 1 | 0.985 |
|  |  | Sex (Male) | 0.219 | 0.183 | 1.192 | 0.233 | 1.422 | 1 | 0.233 |
|  |  | Time of Day (Midday) | -0.212 | 0.093 | -2.268 | 0.023 | 5.224 | 2 | 0.073 |
|  |  | Time of Day (Afternoon) | -0.090 | 0.085 | -1.063 | 0.288 |  |  |  |
|  |  | Herd Size | -0.039 | 0.019 | -2.053 | 0.040 | 4.213 | 1 | 0.04 |
|  |  | Habitat Size | 0.129 | 0.104 | 1.239 | 0.215 | 1.536 | 1 | 0.215 |
| Locomotion | Outdoor | Intercept | -1.923 | 0.150 | -12.828 | <0.001 |  |  |  |
|  |  | Age Class (Subadult) | 0.136 | 0.112 | 1.212 | 0.225 | 1.47 | 1 | 0.225 |
|  |  | Sex (Male) | 0.117 | 0.103 | 1.139 | 0.255 | 1.298 | 1 | 0.255 |
|  |  | Time Category (Midday) | -0.012 | 0.045 | -0.273 | 0.785 | 149.558 | 2 | <0.001 |
|  |  | Time Category (Afternoon) | 0.472 | 0.044 | 10.789 | <0.001 |  |  |  |
|  |  | Temperature | -0.237 | 0.027 | -8.692 | <0.001 | 75.553 | 1 | <0.001 |
|  |  | Herd Size | -0.035 | 0.018 | -1.978 | 0.048 | 3.911 | 1 | 0.048 |
|  |  | Habitat Size | -0.006 | 0.077 | -0.072 | 0.942 | 0.005 | 1 | 0.942 |
|  |  | Weather (Rainy) | -0.234 | 0.118 | -1.992 | 0.046 | 15.841 | 2 | <0.001 |
|  |  | Weather (Sunny) | -0.140 | 0.037 | -3.768 | <0.001 |  |  |  |
|  |  | Public Feeding (Yes) | 0.036 | 0.179 | 0.203 | 0.839 | 0.041 | 1 | 0.839 |
|  | Indoor | Intercept | -2.084 | 0.133 | -15.703 | <0.001 |  |  |  |
|  |  | Age Class (Subadult) | 0.063 | 0.141 | 0.446 | 0.655 | 0.199 | 1 | 0.655 |
|  |  | Sex (Male) | 0.272 | 0.133 | 2.051 | 0.040 | 4.208 | 1 | 0.04 |
|  |  | Time of Day (Midday) | -0.406 | 0.099 | -4.083 | <0.001 | 19.331 | 2 | <0.001 |
|  |  | Time of Day (Afternoon) | -0.254 | 0.086 | -2.935 | 0.003 |  |  |  |
|  |  | Herd Size | -0.040 | 0.014 | -2.794 | 0.005 | 7.804 | 1 | 0.005 |
|  |  | Habitat Size | 0.084 | 0.072 | 1.164 | 0.244 | 1.355 | 1 | 0.244 |
